# Prevalence of colonization with multidrug-resistant bacteria in communities and hospitals in Kenya

**DOI:** 10.1038/s41598-022-26842-3

**Published:** 2022-12-24

**Authors:** Teresa Ita, Ulzii-Orshikh Luvsansharav, Rachel M. Smith, Robert Mugoh, Charchil Ayodo, Beatrice Oduor, Moureen Jepleting, Walter Oguta, Caroline Ouma, Jane Juma, Godfrey Bigogo, Samuel Kariuki, Brooke M. Ramay, Mark Caudell, Clayton Onyango, Linus Ndegwa, Jennifer R. Verani, Susan Bollinger, Aditya Sharma, Guy H. Palmer, Douglas R. Call, Sylvia Omulo

**Affiliations:** 1 Washington State University Global Health-Kenya, Nairobi, Kenya; 2 Division of Healthcare Quality Promotion, U.S. Centers for Disease Control and Prevention, Atlanta, USA; 3 Center for Global Health Research, Kenya Medical Research Institute, Kisumu, Kenya; 4 Center for Microbiology Research, Kenya Medical Research Institute, Nairobi, Kenya; 5 Paul G. Allen School for Global Health, Washington State University, 240 SE Ott Road, Pullman, WA 99164-7090 USA; 6 Center for Health Studies, Universidad del Valle de Guatemala, Guatemala City, Guatemala; 7 Centers for Disease Control and Prevention, Nairobi, Kenya; 8 University of Nairobi Institute of Tropical and Infectious Diseases, Nairobi, Kenya

**Keywords:** Bacteriology, Epidemiology

## Abstract

We estimated the prevalence of extended-spectrum cephalosporin-resistant Enterobacterales (ESCrE), carbapenem-resistant Enterobacterales (CRE), and methicillin-resistant *Staphylococcus aureus* (MRSA) in communities and hospitals in Kenya to identify human colonization with multidrug-resistant bacteria. Nasal and fecal specimen were collected from inpatients and community residents in Nairobi (urban) and Siaya (rural) counties. Swabs were plated on chromogenic agar to presumptively identify ESCrE, CRE and MRSA isolates. Confirmatory identification and antibiotic susceptibility testing were done using the VITEK®2 instrument. A total of 1999 community residents and 1023 inpatients were enrolled between January 2019 and March 2020. ESCrE colonization was higher in urban than rural communities (52 vs. 45%; *P* = 0.013) and in urban than rural hospitals (70 vs. 63%; *P* = 0.032). Overall, ESCrE colonization was ~ 18% higher in hospitals than in corresponding communities. CRE colonization was higher in hospital than community settings (rural: 7 vs. 1%; urban: 17 vs. 1%; with non-overlapping 95% confidence intervals), while MRSA was rarely detected (≤ 3% overall). Human colonization with ESCrE and CRE was common, particularly in hospitals and urban settings. MRSA colonization was uncommon. Evaluation of risk factors and genetic mechanisms of resistance can guide prevention and control efforts tailored to different environments.

## Introduction

Curbing the spread of antimicrobial resistance (AMR) is a global public health priority^1^. In 2019 AMR was associated with 1.3 million deaths, with sub-Saharan Africa having the highest mortality rate attributable to drug-resistant infections^2^. Projections by the Review on Antimicrobial Resistance estimate 10 million deaths per year by 2050 if current trends in AMR continue. The World Bank also forecasts a cumulative economic output loss of USD 100 trillion by 2050^3^. Owing to these projections and estimates, countries around the world have developed National Action Plans following the World Health Organization (WHO)'s guidance^4^. A critical component of these plans is the implementation of robust AMR surveillance systems to detect and quantify AMR as it emerges or spreads. High priorities for these systems include the identification of multidrug-resistant organisms including carbapenem-resistant Enterobacterales (CRE) or extended-spectrum beta-lactamase-producing organisms. These are designated by the US Centers for Disease Control and Prevention (CDC) as high threat organisms because they can cause untreatable or nearly untreatable infections^5^.

In most countries, the detection of these and other organisms by AMR surveillance systems relies on recovery of bacteria from samples collected from patients with clinical infections. In many low- and middle-income countries, this strategy is hampered by limited bacteriology capacity, underuse of clinical laboratories for culture of suspect organisms, limited stocks of laboratory supplies and reagents^6,7^ and poor communication of results and data across sectors. While improving laboratories and diagnostic stewardship practices are critical, other approaches are needed to monitor AMR trends in the near term. One potential approach is to quantify human colonization with antimicrobial-resistant microbes, which may provide a less biased and more complete view of AMR phenotypes and genotypes circulating within a hospital or community^8^. Utilized in this manner, colonization data can serve as useful adjuncts to routine AMR surveillance from clinical samples.

We initiated an Antimicrobial Resistance in Communities and Hospitals (ARCH) study^9^ in Kenya in 2019 to evaluate how colonization data can help assess the diversity and prevalence of antimicrobial-resistant bacteria. Our goal was to estimate the prevalence and describe the epidemiology of human colonization with three clinically relevant, high-threat antimicrobial-resistant organisms: CRE, extended-spectrum cephalosporin-resistant Enterobacterales (ESCrE), and methicillin-resistant *Staphylococcus aureus* (MRSA) in select communities and among patients admitted to the hospitals that serve these communities.

## Methods

### Study locations

Two community sites that have participated in the Population-Based Infectious Disease Surveillance (PBIDS) for over 10 years^10,11^ were selected for this study. Kibera—located in Nairobi County—is an urban informal settlement characterized by poor quality housing, insufficient garbage disposal, lack of formal sewers, and overcrowding (~ 87,000 people/km^2^^12,13^). Participant recruitment was done in the two villages that participate in PBIDS, Gatwekera and Soweto. These villages are divided into 10 clusters for ease of administration. Asembo—located in Siaya County—is a rural community that predominantly practices subsistence farming and fishing, uses domestic waste as agricultural fertilizer, and has households that are organized into dispersed villages^11,13^. Recruitment in Asembo was done in 33 villages.

Study hospitals were selected based on proximity to community sites and the availability of inpatient facilities^9^. Mbagathi County Hospital, which serves Kibera and neighboring communities, is a 300-bed hospital located in Nairobi County with ~ 11,000 annual admissions (2019 hospital estimates). For the Asembo site, three hospitals were included, i.e., Siaya County Referral Hospital, Bondo Sub-county Hospital and St. Elizabeth Lwak Mission Hospital. These had 162, 100 and 50 inpatient beds, and 9000, 2700 and 1300 admissions in 2019, respectively. Of these, St. Elizabeth Lwak Mission Hospital is located within Asembo whereas Bondo sub-county Hospital and Siaya County Referral Hospital are located near the Asembo area, serving patients from Asembo and its environs.

### Sampling

Sampling in communities and hospitals was done between January 2019 and March 2020. Our target sample sizes were 768 adults and 768 children at community sites, based on a design effect of 2, household completion proportion of 75%, a relative precision of 20%, and an average household size of 4.2. A similar approach was used to generate a target sample size of 509 for the hospitalized population. Two-thousand households were randomly selected from a list of PBIDS households in Kibera (n = 4598) and in Asembo (n = 7790). These were then grouped by cluster (in Kibera) or village (in Asembo). Within consenting households, a child < 5 years and an adult ≥ 18 years (assumed to have different exposures and widely varying risk factors for colonization with the target organisms^14^) were selected at random from among age-eligible household members. Selected participants were further screened to exclude individuals who had not consistently resided in the household for at least four weeks prior to the study, and those with fever, diarrhea, or cough on the day of the visit. Clusters/villages were visited every Monday to Thursday to enroll new households, and each cluster/village was visited at least once every two weeks to minimize temporal differences that might influence prevalence estimates.

Probability proportional to size sampling (based on the number of beds) was used to determine the number of inpatients to enroll. Simple random sampling was then applied by first developing a serialized list of inpatient beds for each ward and then randomly selecting five inpatients from this list for enrollment each morning. At the urban site, all 509 inpatients were enrolled from Mbagathi County Hospital. However, for the rural site, the total number of hospitalized participants to enroll per hospital was determined by dividing the number of serialized beds in each hospital by the total number of beds across the three hospitals and multiplied by 509. All patients, regardless of age, were considered eligible for random selection (patients < 18 years were considered children). Patients were excluded if they had severe neutropenia (absolute neutrophil count < 500 cells/µL of blood) and gastrointestinal bleeding documented in their medical records or if they had been enrolled in a previous sampling round.

### Data collection

During community enrollment, informed consent was first obtained from the household head. A questionnaire was then administered to collect demographic data about household members and household characteristics. Informed consent was then obtained from the adult participant to be enrolled. To enroll a child, consent was obtained from the household head or the child’s primary caretaker. At hospital sites, informed consent was obtained from hospitalized patients aged ≥ 18 years, or from guardians of patients who were unconscious, sedated or < 18 years. Informed assent was also obtained from children aged between 7 and 17 years. Data on patient demographics were abstracted from medical records.

### Laboratory methods

The methods used for sample collection and processing in this study have previously been published^9^. Briefly, nasal and rectal swabs (ESwab, Copan diagnostics, CA) were collected from participants and placed in Amies transport media then labeled using barcodes. All samples were transported at 4 °C to the laboratory for same-day processing. Where stool was collected in lieu of a rectal swab, the sample was converted to a swab at the lab, placed in transport media and barcoded. Nasal samples were cultured on HardyCHROM™ MRSA agar plates. Stool/rectal samples were cultured on HardyCHROM™ CRE agar plates and HardyCHROM™ ESBL agar plates. All HardyCHROM agar plates were ordered pre-made from the manufacturer and each batch was tested before use with manufacturer-recommended quality control strains from American Type Culture Collection (ATCC; www.atcc.org).

After incubation at 37 °C for 18–24 h, up to three pink colonies of different morphologies were selected as presumptive MRSA from nasal swabs (MRSA plates) or *Escherichia coli* from stool/rectal swabs (CRE and ESBL plates). Bluish-green colonies, presumptively identified as *Klebsiella* sp*.*, *Enterobacter* sp., *Serratia* sp. or *Citrobacter* sp. were also collected from stool/rectal swab culture plates. Once purified via subculture, confirmation of bacterial species identity and antibiotic resistance phenotypes were determined using a VITEK®2 instrument following manufacturer instructions. Quality control testing for the VITEK®2 cards ID and antimicrobial susceptibility testing (AST) (GN-71 and GP-75) was done using manufacturer recommended ATCC strains. AST breakpoints were interpreted based on CLSI M100 guidelines^15^; breakpoint classifications are provided in Supplementary Table S1.

### Definitions

Isolates were classified as ESCrE if susceptible to all carbapenems tested (ertapenem, meropenem and imipenem) and non-susceptible (intermediate or resistant) to ceftriaxone. Those classified as CRE were non-susceptible to ≥ 1 carbapenem, while MRSA isolates exhibited full resistance to oxacillin and/or had a positive cefoxitin screen test on the VITEK®2. Isolates were defined as multidrug-resistant if they were non-susceptible to at least one agent in ≥ 3 antimicrobial classes^16^. Isolates that were non-susceptible to the three carbapenems, beta-lactam and fluoroquinolones were considered to represent “difficult-to-treat resistance”, a definition described by Kadri et al. in a recent Clinical Infectious Disease paper^17^.

### Statistical methods

We accounted for potential design effects from the two-stage cluster sampling used at the community sites, which differed from the simple random sampling design used in hospital enrollment patients. Due to these design differences, comparisons between these two data sets were considered significantly different when 95% confidence intervals for point estimates did not overlap.

The community sampling design included households as the primary sampling unit, stratified by rural and urban settings. Individuals were treated as secondary elements. Probability weights for the first sampling stage were based on the number of enrolled households (n) and the total households participating in PBIDS (N). Probability weights for the secondary elements were calculated at the household level by dividing the number of individuals enrolled (n) by the total number of eligible individuals within the household (N) for each age category (adults and children). The *svyset* command in Stata v.17^18^ was used to specify the study design including the probability weights and strata. To generate weighted prevalence estimates and confidence intervals, and to compare mean colonization across location and age group at the community site (using *tab* command), the svy prefix was used. Design effects were determined by dividing the variance of the prevalence estimates under the sampling design by the variance of estimates from a hypothesized simple random sample of the same size (using the *estat effects* command in Stata v.17).

The prevalence of ESCrE, CRE or MRSA colonization at hospital sites was calculated by dividing the number of individuals from whom one or more of these isolates were recovered by the total number of participant samples (stratified by age group). The chi-square test was used to compare prevalence of colonization with ESCrE, stratified by age. Sample size limitations prevented age stratification for CRE and MRSA colonization data.

Antibiotics were categorized into classes (Supplementary Table S1) and the distribution of isolates examined relative to multidrug resistance (MDR; pooled across adults and children). A Wilcoxon rank-sum test was used to test the hypothesis that the observed resistance to antibiotic classes for community isolates was different from that observed in hospital isolates. Heatmaps^19^ and complex UpsetR^20,21^ were used to generate figures summarizing antibiotic susceptibility results.

### Ethical approval

This study was approved by the ethics and research committee at the Kenyatta National Hospital/University of Nairobi (ERC #P164/03/2018), with reliance approval from Washington State University (IRB# 16742-001), the Kenya Medical Research Institute and the Centers for Disease Control and Prevention (#7111). This project was licensed by the Kenya National Commission for Science, Technology & Innovation (NACOSTI-P-21-12461). Research and administrative approvals were also sought from the Nairobi and Siaya county governments and from the boards of individual hospitals. All research was performed in accordance with relevant guidelines and regulations.

## Results

A total of 1999 participants were enrolled at community sites, including 1090 participants in Kibera (urban) and 909 in Asembo (rural). Adult participants were primarily female (78%, n = 1476) with a median age of 35 years [range: 17–92]. A slight majority of child participants were male (52%, n = 523), with a median age of 2 years [0–5]. At the hospital sites, 1023 participants were enrolled. Of these, 510 were enrolled at Mbagathi hospital (urban) and 513 in Siaya county hospitals (rural). The median number of hospitalization days prior to enrollment was five [0–64] for participants at Mbagathi hospital and three [0–79] at Siaya hospitals. Enrolled adults were predominantly female (68%, n = 614) with a median age of 35 years [18–89] while children were mostly male (54%, n = 409) with a median age of 1 year [0–17].

All except two participants enrolled at community sites (n = 1995) provided a nasal swab and 1715 (86%) provided a stool sample (Supplementary Fig. S1). All 1023 participants enrolled at hospital sites provided nasal swabs and 852 (83%) provided a rectal swab or stool sample. In total 3177 isolates were recovered from community (1725) and hospital samples (1425) following culture on HardyCHROM™ agar plates (2179 ESCrE, 596 CRE, and 402 MRSA). Confirmatory identification by the VITEK®2 Compact yielded 1965 isolates with AMR phenotypes of interest including 1015 ESCrE, 23 CRE, and 23 MRSA isolates from community sites, and 772 ESCrE, 102 CRE, and 30 MRSA isolates from hospital sites. Based on the definitions employed for this study (see methods), the correspondence between agar results and VITEK®2 results were 82% (ESCrE), 21% (CRE) and 13% (MRSA). All analyses were limited to VITEK®2-confirmed isolates.

### Prevalence of ESCrE-, CRE- and MRSA-colonized participants

Design effects attributable to the two-stage sampling design used in communities ranged from 1.06 to 1.35 for estimates of ESCrE colonization (Table 1). The ranges for estimated design effect for CRE and MRSA varied widely (including < 1.0), consistent with non-asymptotic variance estimates that can be expected with small numbers of colonized individuals (range 0–17 participants depending on category, Table 1).

**Table 1:** Prevalence of colonization with antimicrobial-resistant bacteria in rural and urban communities, Kenya.

	Rural community			Urban community			*P*-value^d^
	%^a^	95% CI (n/N)^b^	DEFF^c^	%	95% CI (n/N)	DEFF^c^	
**ESCrE**^e^							
Adult	44	40–49 (283/634)	1.16	51	47–55 (346/673)	1.29	**0.027**
Child	46	37–54 (74/155)	1.20	53	46–60 (135/253)	1.06	0.18
Overall	45	41–49 (357/789)	1.28	52	48–56 (481/926)	1.35	**0.013**
**CRE**							
Adult	1	1–2 (7/634)	1.06	2	1–3 (15/673)	1.02	–
Child	0	– (0/155)	–	0	0–3 (1/253)	0.84	–
Overall	1	0–2 (7/789)	1.10	1	1–3 (16/926)	1.14	–
**MRSA**							
Adult	1	0–2 (5/705)	0.93	2	1–2 (12/769)	1.13	–
Child	0	0–3 (1/201)	0.86	2	1–4 (5/320)	0.86	–
Overall	1	0–2 (6/906)	1.33	2	1–3 (17/1,089)	1.68	–

Prevalence estimates, confidence intervals and statistical tests account for multi-stage cluster sampling design.

^a^Prevalence is calculated on a per person level. “–” value not calculated due to limited sample size.

^b^CI = confidence interval (n is the number of individuals colonized with ESCrE, CRE or MRSA bacteria, and N is the total number of people sampled).

^c^DEFF = design effect, which is the ratio of the variance of the prevalence estimate when accounting for sampling design effects to the variance of the prevalence estimate assuming a simple random design. If DEFF = 1.0, there is no design effect.

^d^*P*-value for rural versus urban comparisons.

^e^ESCrE = extended-spectrum cephalosporin-resistant Enterobacteriaceae; CRE = carbapenem-resistant Enterobacteriaceae; MRSA = Methicillin-resistant *Staphylococcus aureus*. Significant values are in bold.

The overall prevalence of ESCrE-colonization among community participants was significantly lower for rural vs. urban sites (45 and 52%, respectively, *P* = 0.013, Table 1), and significantly lower for rural hospitals compared with urban hospitals (63 and 70%, respectively, *P* = 0.032, Table 2). ESCrE prevalence was 18% higher in the rural hospitals compared to the rural community with non-overlapping 95% confidence intervals (Tables 1 and 2). ESCrE prevalence was 18% higher in the urban hospital compared to the urban community with non-overlapping 95% confidence intervals (Tables 1 and 2).

**Table 2:** Prevalence of colonization with antimicrobial-resistant bacteria in rural and urban hospitals, Kenya.

	Rural hospitals		Urban hospital		*P*-value^c^
	%^a^	95% CI (n/N)^b^	%	95% CI (n/N)	
**ESCrE**^d^					
Adult	64	58–70 (189/295)	67	59–75 (107/159)	0.49
Child	61	53–68 (109/180)	72	65–78 (156/218)	**0.021**
Overall	63	58–67 (298/475)	70	65–74 (263/377)	**0.032**
**CRE**					
Overall	7	5–9 (31/475)	17	13–21 (63/377)	**< 0.001**
**MRSA**					
Overall	3	2–5 (15/513)	3	2–5 (15/510)	0.99

Prevalence estimates, confidence intervals and statistical tests assumed a simple random design.

^a^Prevalence is calculated on a per person level.

^b^CI = confidence interval (n is the number of individuals colonized with ESCrE, CRE or MRSA bacteria, and N is the total number of people sampled).

^c^*P*-value for comparisons between participants in rural vs. urban hospitals.

^d^ESCrE = extended-spectrum cephalosporin-resistant Enterobacteriaceae; CRE = carbapenem-resistant Enterobacteriaceae; MRSA = Methicillin-resistant *Staphylococcus aureus*. Significant values are in bold.

When examined by age group and stratified by community, hospital, urban and rural, prevalence estimates for ESCrE differed by < 5% between adults and children (Tables 1 and 2). Adults in the urban community had a statistically higher ESCrE prevalence (51%) compared to those in the rural community (44%; *P* = 0.027, Table 1). Children in urban hospitals had a statistically greater colonization rate (72%) compared with children in rural hospitals (61%; *P* < 0.021, Table 2). The prevalence of CRE was < 2% in urban and rural communities (Table 1) but was significantly higher in urban hospitals (17%) compared to rural hospitals (7%) (*P* < 0.001, Table 2). The 95% confidence intervals did not overlap for the overall prevalence of CRE in rural and urban hospitals when compared to their respective communities (Tables 1 and 2). MRSA prevalence was < 3% regardless of setting and all point estimates had overlapping 95% confidence intervals. CRE and MRSA prevalence estimates were not stratified by age due to limited sample sizes.

### Characteristics of ESCrE isolates

**Figure 1:**
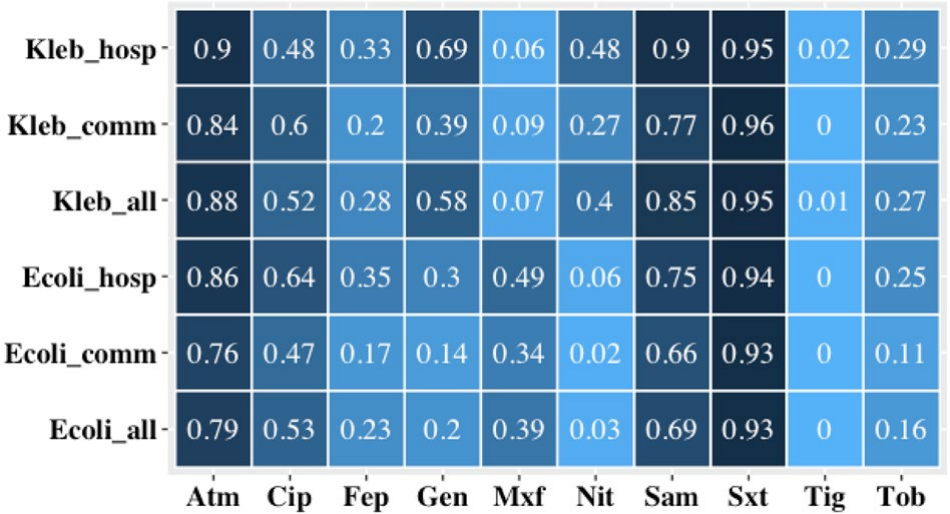
Heatmap showing the proportion of resistant isolates among ESCrE for ten of the seventeen antibiotics tested. Increasing color intensity denotes increasing proportion of resistant isolates. Seven are not shown because all isolates were fully resistant to ceftriaxone, ampicillin, and cefazolin and none had intermediate or complete resistance to amikacin, ertapenem, imipenem and meropenem. Heatmap includes 1345 *E. coli* (hospital = 492, community = 863) and 442 *Klebsiella oxytoca* and *K. pneumoniae* pooled (hospital = 280 hospital, community = 162). Antibiotics: aztreonam (Atm), ciprofloxacin (Cip), cefepime (Fep), gentamicin (Gen), moxifloxacin (Mxf), nitrofurantoin (Nit), ampicillin/sulbactam (Sam), sulfamethoxazole/trimethoprim (Sxt), tigecycline (Tig), and tobramycin (Tob).

A total of 1787 ESCrE isolates (1345 *E. coli* and 442 *Klebsiella* sp.) were characterized for antibiotic resistance phenotypes from 1336 participants (Fig. 1 and Supplementary Fig. S2). Of these, 1785 (99%) were resistant to ceftriaxone (MIC ≥ 4 µg/ml, Supplementary Table S1) and all were resistant to ampicillin and cefazolin. Resistance to ampicillin/sulbactam, aztreonam, ciprofloxacin and sulfamethoxazole/trimethoprim was > 50% among the isolated species (Fig. 1). No isolate had intermediate or complete resistance to amikacin. In general, the prevalence of resistance was higher among *Klebsiella* sp. than *E. coli* isolates except in the case of moxifloxacin. *Klebsiella* sp. isolates from hospital settings harbored greater resistance to gentamicin (69%) and nitrofurantoin (48%) compared with community isolates (39% and 27%, respectively).

When grouped into resistant phenotypes, < 1% of ESCrE isolates were resistant to ceftriaxone alone. More than half (54%) of hospital isolates were resistant to ≥ 6 six antibiotics (including ceftriaxone) compared with 29% among community isolates (Supplementary Fig. S2). A total of 81 phenotype combinations (intersections) were identified among *Klebsiella* sp. (n = 442) and 88 among *E. coli* (n = 1345). Most (56%) *Klebsiella* sp. isolates and 33% of *E. coli* isolates were resistant to ≥ 6 six antibiotics.

### Characteristics of CRE isolates

**Figure 2:**
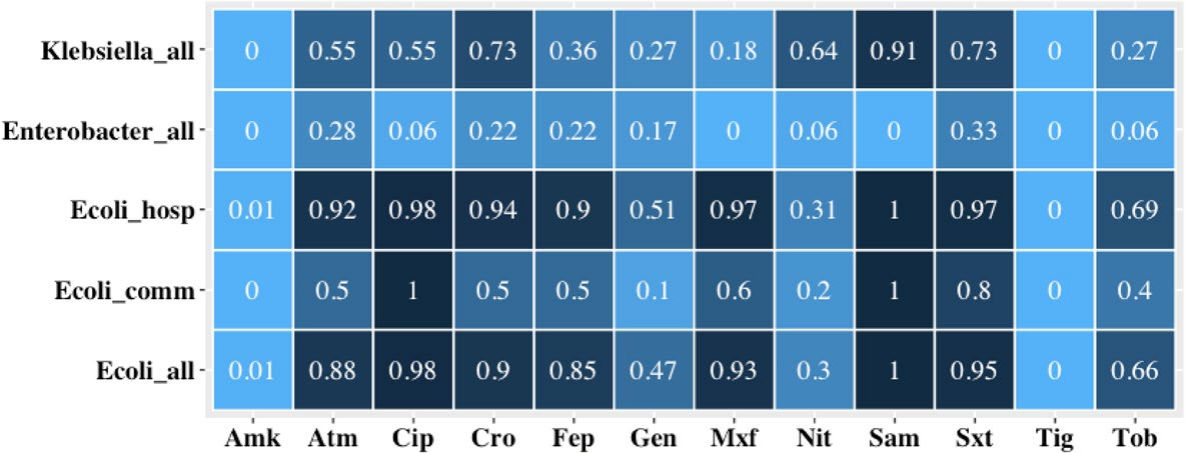
Heatmap showing the proportion of resistant isolates among CRE for 12 of the 17 antibiotics tested. Increasing color intensity denotes increasing proportion of resistant isolates. The three carbapenems tested (ertapenem, imipenem and meropenem) are not shown. Ampicillin and cefazolin are also not shown because isolates were fully resistant. Heatmap includes 96 *E. coli* (hospital = 86, community = 10), 11 *Klebsiella oxytoca* and *K. pneumoniae* pooled, and 18 *Enterobacter aerogenes* and *E. cloacae* pooled. Antibiotics: amikacin (Amk), aztreonam (Atm), ciprofloxacin (Cip), ceftriaxone (Cro), cefepime (Fep), gentamicin (Gen), moxifloxacin (Mxf), nitrofurantoin (Nit), ampicillin/sulbactam (Sam), sulfamethoxazole/trimethoprim (Sxt), tigecycline (Tig), and tobramycin (Tob).

In total, 125 CRE isolates (96 *E. coli*, 11 *Klebsiella* sp., and 18 *Enterobacter* sp.) were characterized phenotypically from 119 individuals. All isolates were resistant to ampicillin and cefazolin and 102 (82%) were fully resistant to ≥ 1 carbapenem (Supplementary Fig. S3). Except for nitrofurantoin, resistance to individual antibiotics was highest among *E. coli* (especially among hospital samples) followed by *Klebsiella* sp. then *Enterobacter* sp. (Fig. 2). Half (51%) of all *E. coli* isolates were fully resistant to the three carbapenems tested, while 21% were resistant to ertapenem only (Supplementary Fig. S3). Comparatively, 18% of *Klebsiella* sp. isolates were fully resistant to the three carbapenems while 36% were resistant to ertapenem only. Among *Enterobacter* sp. isolates, 22% were fully resistant to all three carbapenems (22%), while 28 and 44% showed full and intermediate resistance, respectively, to imipenem alone (Supplementary Fig. S3). All *E. coli* and *Klebsiella* sp. isolates were multidrug-resistant compared with 56% of *Enterobacter* sp. Isolates (Supplementary Fig. S4). The majority (74%) of *E. coli* isolates were resistant to ≥ 6 antibiotic combinations compared to 45% and 18% among *Klebsiella* sp. and *Enterobacter* sp. isolates, respectively (*P* < 0.05).

Of the 125 CRE strains identified, 49 (39%) met the “difficult-to-treat resistance” definition and were all *E. coli*. Seven of these were intermediate to tigecycline. For comparative analyses, we considered the first isolate identified when more than one CRE was identified per participant allowing for 119 independent observations. Forty-eight isolates were classified as difficult-to-treat resistance phenotypes. These isolates were more likely to be found among urban than rural participants (*P* = 0.028), but did not vary significantly when compared between communities and hospitals (*P* = 0.09) or adults and children (*P* = 0.54).

### Characteristics of MRSA isolates

Fifty-three MRSA isolates were collected from 51 participants, of which 40% were resistant to oxacillin and had a positive cefoxitin screen (Supplementary Fig. S5). The remaining isolates were classified either by their resistance to oxacillin (32%) or a positive result for the cefoxitin screen (28%; Supplementary Fig. S5). All isolates tested susceptible to linezolid, tigecycline and vancomycin, while 8% were resistant to daptomycin.

## Discussion

For the populations included in the current study, the prevalence of colonization with ESCrE was high (> 40%) across all populations tested (urban/rural, hospital/community, and adult/child). While CRE prevalence was low (< 3%) in community settings, the prevalence observed among hospitalized patients (7–17%) was concerning. Newer antibiotic therapies against CRE infections are limited in Kenya^22^ and 39% of CRE isolates met the “difficult-to-treat resistance” definition, which has been associated with poor clinical outcomes when such organisms cause invasive infections^17^. MRSA colonization rates were very low, consistent with earlier, smaller studies in Kenya^23,24^.

The largest differences in colonization prevalence across ESCrE, CRE and MRSA were observed between hospital and community settings. Hospital settings had consistently higher AMR prevalence compared with communities, regardless of location or participant age groups. Additionally, even within AMR classes, organisms recovered in hospitals were resistant to more antibiotic classes than those in the community. Smaller, but consistent, differences in AMR prevalence were observed when comparing urban and rural locales. Urban populations harbored a higher prevalence of ESCrE and CRE compared with rural populations when compared within hospital and community settings. AMR prevalence did not differ significantly when adults and children were analyzed within settings.

The difference in AMR prevalence between hospitals and communities may be related to widespread use of broad-spectrum antibiotics, such as ceftriaxone, among inpatients^25,26^. Additionally, poor infection prevention and control (IPC) practices can promote the transmission of antimicrobial-resistant organisms. While improving antibiotic stewardship and IPC in Kenyan hospitals is an important goal, the high ESCrE colonization prevalence among community residents in this study indicates that focusing solely on hospitals as intervention sites for AMR prevention and control may be insufficient. Identifying the drivers of acquisition and patterns of persistence and clearance in hospitals and communities is as a necessary step to develop and prioritize interventions that reduce colonization with resistant organisms. Prior studies in Kenya and East Africa suggest that focusing on outpatient antibiotic prescribing practices, restricting access to antibiotics without prescription, regulating antibiotic use in animal husbandry, and improving sanitation and hygiene may be important areas for intervention^27–30^.

The higher prevalence of ESCrE and CRE colonization among urban than rural participants may be related to several factors, including population density, access to antibiotics and healthcare utilization. While rural and urban risk factors for AMR prevalence differ within hospital and community spheres, a robust analysis of the drivers of AMR within these settings is needed to guide the development of targeted interventions for AMR prevention and control^31,32^.

Data on colonization with antimicrobial-resistant bacteria from Kenya are not included in the most recent WHO Global Antimicrobial Resistance and Use Surveillance System report for 2021^33^, nor are national estimates of antibiotic resistance available. Consequently, we cannot compare data from our study with routine AMR surveillance data. However, existing data on prevalence of ESCrE, or ESBL-producing Enterobacterales, CRE and MRSA in Kenya are available from published reports^24,34^, although these are limited to studies of infections among hospitalized patients and have small sample sizes. Estimates of prevalence in these studies vary widely. One study of *E. coli* isolated from urine cultures of symptomatic patients identified ESBL-producers in 25% of the 94 samples tested^35^. Other studies have reported estimates ≥ 50% of Enterobacterales isolates classified as ESBL-producers among hospitalized patients, which mirror the high prevalence of colonized inpatients found in this study^36,37^. Fewer studies have evaluated carbapenem resistance, but those that have report CRE prevalence estimates ranging from 6.5–25% among hospitalized patients^36,37^. MRSA studies among hospitalized patients have also reported estimates of 3.7% among select private hospitals in Kenya^38^ to 53% at the Kenyatta National Hospital^37,39^, a national referral hospital. These varied estimates from mostly single-center studies with small sample sizes demonstrate the value of robust AMR surveillance methodology, including assessment of colonization, to accurately define the burden of AMR and to track AMR changes, emergence, and effects of interventions.

A potential limitation of this study was our inability to determine if colonizing organisms harboring extended-spectrum cephalosporin resistance or carbapenem resistance were pathogenic. Indeed, some of the detected isolates may be commensal organisms with low likelihood of causing invasive infections in people. However, even non-pathogenic organisms that are less clinically important on an individual level have the potential to contribute to the spread of AMR via mobile genetic elements^40^, especially within hospital environments^41^. Further evaluation of pathogenicity or triangulation with other studies at these facilities or areas is important to qualify these prevalence results. While the low detection levels for MRSA and CRE (particularly in community settings) are encouraging, the small numbers likely limited our power to detect differences across the enrolled populations.

There is also a possibility that the point estimates for colonization in this study may be biased by the selective media used for initial isolation of bacteria. Using a VITEK®2 instrument as the gold standard for antibiotic susceptibility testing, a relatively high percentage (82%) of isolates selected from HardyCHROM™ ESBL agar had corresponding ESCrE phenotypes. The correspondence for the CRE media (21%) and MRSA media (13.2%) was much lower. It is possible that poor correspondence between initial selective isolation and final phenotypic classification could lead to biased point estimates, but a different standard would be needed in parallel with HardyCHROM™ media to ascertain the diagnostic sensitivity and specificity of these reagents (e.g., it is possible that HardyCHROM™ reagents have very high diagnostic sensitivity but low diagnostic specificity, in which case bias would be minimal).

In conclusion, we found a high prevalence of highly resistant Gram-negative organisms colonizing persons across a range of locations and ages in Kenya, including community dwellers. Robust AMR surveillance data, including colonization data as shown in this study, are critical to evaluating AMR trends and response to interventions. Identifying risk factors and resistance genotypes can improve our understanding of these data and guide the development and implementation of IPC measures in different environments.

### CDC Disclaimer

The findings and conclusions in this report are those of the authors and do not necessarily represent the official position of the US Centers for Disease Control and Prevention.

## Supplementary Information

Supplementary Information.

## Data Availability

We have provided minimal anonymized data as a file under the supporting information.

## Abbreviations

AMR: Antimicrobial resistance
AST: Antimicrobial susceptibility testing
ATCC: American Type Culture Collection
CDC: Centers for Disease Control and Prevention
CRE: Carbapenem-resistant Enterobacterales
DEFF: Design effect
ESBL: Extended-spectrum beta-lactamase
ESCrE: Extended-spectrum cephalosporin-resistant Enterobacterales
IPC: Infection prevention and control
MDRO: Multidrug-resistant organism
MIC: Minimum inhibitory concentration
MRSA: Methicillin resistant *Staphylococcus aureus*
PBIDS: Population-Based Infectious Disease Surveillance
WHO: World Health Organization
