# Supplementary material for: Prevalence of colonization with multidrug-resistant bacteria in communities and hospitals in Kenya
Source: Sci Rep. 2022 Dec 24;12:22290. doi: 10.1038/s41598-022-26842-3 (PMC9789952; doi:10.1038/s41598-022-26842-3)
Supplement: Supplementary file 1 — Supplementary Information. [file 41598_2022_26842_MOESM1_ESM.docx]

**Prevalence of colonization with multidrug-resistant bacteria in communities and hospitals in Kenya**

Teresa Ita^1^*, Ulzii-Orshikh Luvsansharav^2^*, Rachel M. Smith^2^*, Robert Mugoh^1^, Charchil Ayodo^1^, Beatrice Oduor^1^, Moureen Jepleting^1^, Walter Oguta^1^, Caroline Ouma^3^, Jane Juma^3^, Godfrey Bigogo^3^, Samuel Kariuki^4^, Brooke M. Ramay^5,6^, Mark A. Caudell^5^, Clayton Onyango^7^, Linus Ndegwa^7^, Jennifer R. Verani^7^, Susan Bollinger^2^, Aditya Sharma^2^, Guy H. Palmer^1,5,8^, Douglas R. Call^5^, and Sylvia Omulo^1,5,8**^

^1^Washington State University Global Health-Kenya, Nairobi, Kenya, ^2^Division of Healthcare Quality Promotion, U.S. Centers for Disease Control and Prevention, Atlanta, USA, ^3^Center for Global Health Research, Kenya Medical Research Institute, Kisumu, Kenya, ^4^Center for Microbiology Research, Kenya Medical Research Institute, Nairobi, Kenya, ^5^Paul G. Allen School for Global Health, Washington State University, Pullman, WA, USA, ^6^Center for Health Studies, Universidad del Valle de Guatemala, ^7^Centers for Disease Control and Prevention, Nairobi, Kenya, ^8^University of Nairobi Institute of Tropical and Infectious Diseases.

Running head: Multidrug resistance in Kenya

*Co-first authors

**Corresponding author:

Sylvia Omulo

240 SE Ott Road

Pullman, WA 99164-7090

Tel: 509-335-6328

sylvia.omulo@wsu.edu

# Supplementary information

**Supplementary Table S1**. **List of antibiotics tested and their interpretive criteria**. ^Agent has the potential to concentrate at an anatomical site, *CLSI 2020 breakpoints unavailable, Vitek AST GN-71 and GP-75 ranges applied.

| **Vitek2 cards** | **Antibiotic class** | **Antibiotic code** | **Antibiotic name** | **Interpretive categories and MIC breakpoints** | | |
| --- | --- | --- | --- | --- | --- | --- |
|  |  |  |  | **Susceptible (S)** | **Intermediate (I)** | **Resistant (R)** |
| GN71 card | Aminoglycosides | AMK | Amikacin | <=16 | 32^ | >=64 |
|  |  | GEN | Gentamicin | <=4 | 8^ | >=16 |
|  |  | TOB | Tobramycin | <=4 | 8^ | >=16 |
|  | Beta-lactam inhibitor | SAM | Ampicillin/Sulbactam | <=8/4 | 16/8^ | >=32/16 |
|  | Carbapenems | ETP | Ertapenem | <=0.5 | 1 | >=2 |
|  |  | IMP | Imipenem | <=1 | 2 | >=4 |
|  |  | MEM | Meropenem | <=1 | 2 | >=4 |
|  | Non-extended spectrum cephalosporins | CEZ | Cefazolin | <=2 | 4 | >=8 |
|  | Extended spectrum cephalosporins | CRO | Ceftriaxone | <=1 | 2^ | >=4 |
|  |  | FEP | Cefepime | <=2 | - | >=16 |
|  | Monobactams | ATM | Aztreonam | <=4 | 8^ | >=16 |
|  | Nitrofuran | NIT | Nitrofurantoin | <=32 | 64 | >=128 |
|  | Penicillins | AMP | Ampicillin | <=8 | 16^ | >=32 |
|  | Quinolones | CIP | Ciprofloxacin | <=0.25 | 0.5^ | >=1 |
|  |  | MXF | Moxifloxacin | <=0.25* | - | >=8* |
|  | Sulphonamides | SXT | Sulfamethoxazole/Trimethoprim | <=238 | - | >=4/76 |
|  | Tetracyclines | TGC | Tigecycline | <=0.5* | - | >=8* |
| GP75 card | Aminoglycosides | GEN | Gentamicin | <=4 | 8 | >=16 |
|  | Antimycobacterials | RIF | Rifampicin | <=1 | 2 | >=4 |
|  | Glycopeptide | VAN | Vancomycin | <=2 | 4-8 | >=16 |
|  | Glycylcyclines | TGC | Tigecycline | 0.12* | - | 2* |
|  | Lincosamides | CLI | Clindamycin | <=0.5 | 1-2 | >=4 |
|  | Lipopeptides | DAP | Daptomycin | 0.12* | - | 8* |
|  | Macrolide | ERY | Erythromycin | <=0.5 | 1-4 | >=8 |
|  | Nitrofuran | NIT | Nitrofurantoin | <=32 | 64 | >=128 |
|  | Oxazolidinones | LNZ | Linezolid | <=4 | - | >=8 |
|  | Penicillins | OX1 | Oxacillin | <=2 | - | >=4 |
|  | Quinolones | CIP | Ciprofloxacin | <=1 | 2 | >=4 |
|  |  | LEV | Levofloxacin | <=1 | 2 | >=4 |
|  |  | MXF | Moxifloxacin | <0.5 | 1 | >=2 |
|  | Sulphonamides | SXT | Sulfamethoxazole/Trimethoprim | <=2/38 | - | >=4/76 |
|  | Tetracyclines | DOX | Doxycycline | <=4 | 8 | >=16 |
|  |  | TET | Tetracycline | <=4 | 8 | >=16 |

| **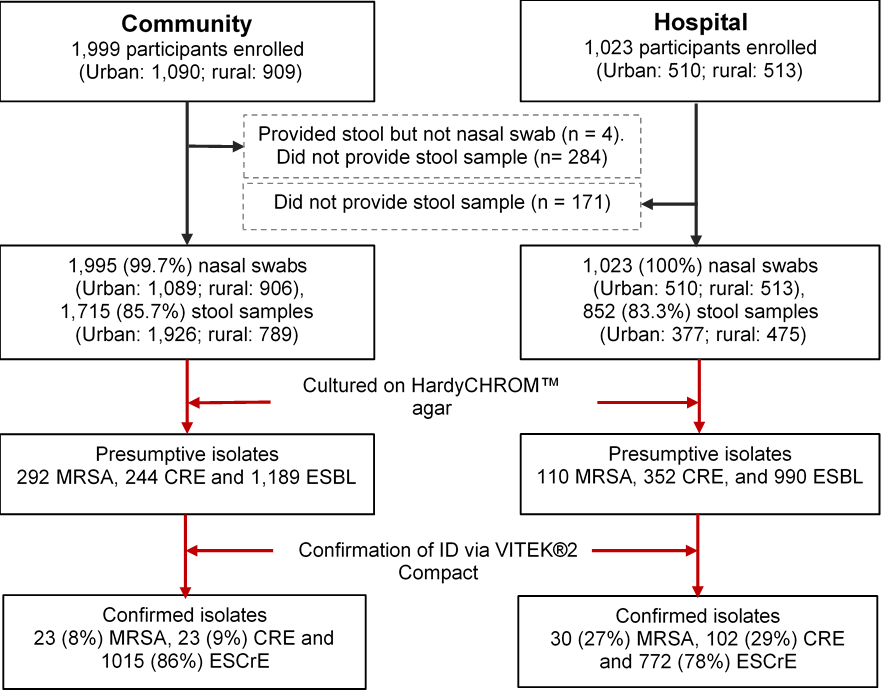** |
| --- |

**Supplementary Fig. S1.** **Summary of enrollment, sample collection and bacterial isolation numbers at community and hospital sites**. CRE: Carbapenem-resistant Enterobacteriaceae; ESBL: Extended-spectrum beta-lactamase-producing Enterobacteriaceae; ESCrE: Extended-spectrum cephalosporin-resistant Enterobacteriaceae; MRSA: Methicillin-resistant *Staphylococcus aureus*.

| 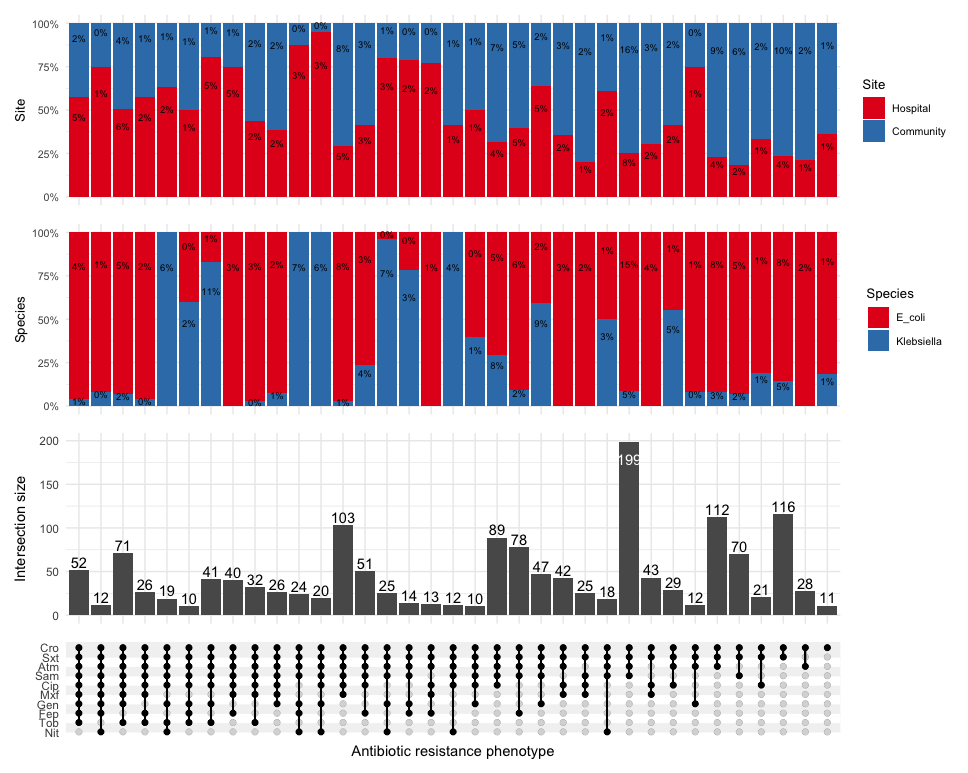 |
| --- |

**Supplementary Fig. S2. ESCrE isolates categorized by multidrug resistance phenotype (MDR).** Lower panel shows the MDR intersections for tested antibiotics. The bars show the number of isolates with the depicted MDR phenotype. Middle panel shows the percent of total *E. coli* (n = 1,345) and total *Klebsiella* sp. (n = 442) corresponding to each MDR phenotype (0% corresponds to < 0.5%). Top figure shows the percent distribution of ESCrE MDR isolates for hospitals (n = 772) and community samples (n = 1,015) (values do not add up to 100% because only intersections with >=10 isolates are shown here). Antibiotics: ceftriaxone (Cro), sulfamethoxazole+trimethorim (Sxt), aztreonam (Atm), ampicillin+sublactam (Sam), ciprofloxacin (Cip), moxifloxacin (Mxf), gentamicin (Gen), cefepime (Fep), tobramycin (Tob), and nitrofurantoin (Nit).

| 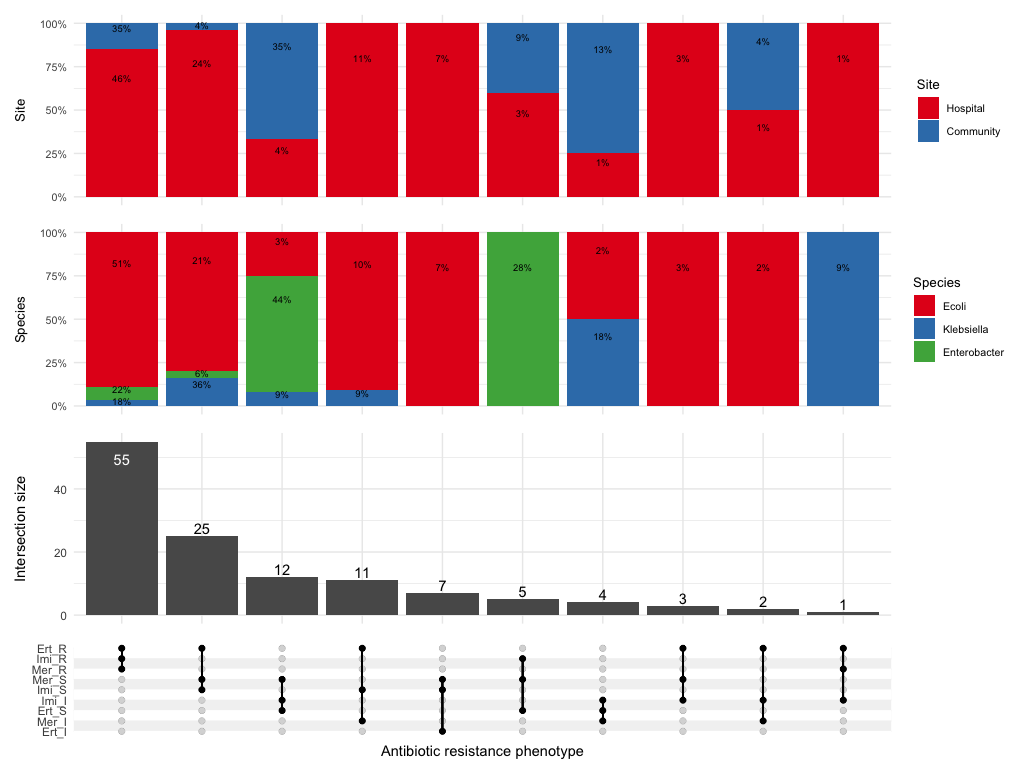 |
| --- |

**Supplementary Fig. S3.** **Distribution of CRE phenotypes [sensitive(S), intermediate (I), resistant (R)] for the three tested carbapenems**. The bars show the number of isolates with the depicted MDRO phenotype (intersection). Middle figure shows the percent of total *E. coli* (n = 96) *Klebsiella* sp. (n = 11) and *Enterobacter* sp. (n = 18) corresponding to each resistance phenotype. Top figure shows the percent distribution phenotypes isolates for hospitals (n = 102) and communities (n = 23). Antibiotics: ertapenem (Ert), imipenem (Imi), and meropenem (Mer).

| 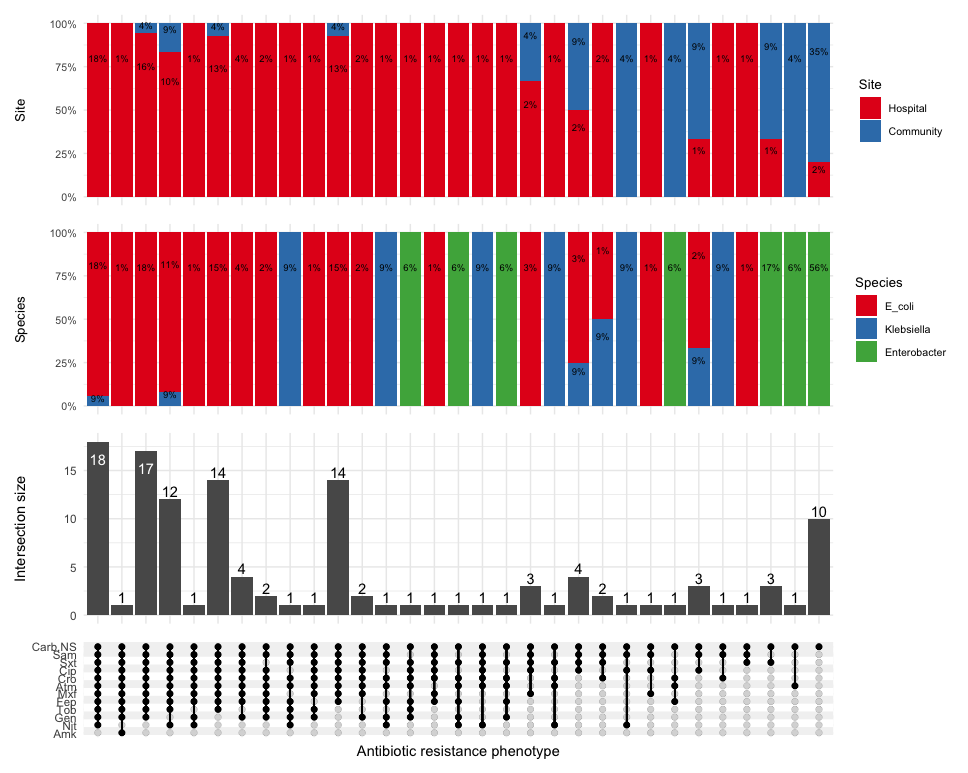 |
| --- |

**Supplementary Fig. S4**. **CRE isolates categorized by multidrug resistance phenotype (MDR)**. Lower panel shows the MDR intersections for all cases for which >= 1 isolates were observed. The bars show the number of isolates with the depicted MDRO phenotype. Middle figure shows the percent of total *E. coli* (n = 96) *Klebsiella* sp. (n = 11) and *Enterobacter* sp. (n = 18) corresponding to each MDRO phenotype (0% corresponds to < 0.5%). Top figure shows the percent distribution of CRE MDRO isolates for hospitals (n = 102) and communities (n = 23). Antibiotics: non-sensitive to carbapenems (Carb. NS), ampicillin+sublactam (Sam), sulfamethoxazole+trimethorim (Sxt), ciprofloxacin (Cip), ceftriaxone (Cro), aztreonam (Atm), moxifloxacin (Mxf), cefepime (Fep), tobramycin (Tob), gentamicin (Gen), nitrofurantoin (Nit), and amikacin (Amk).

| 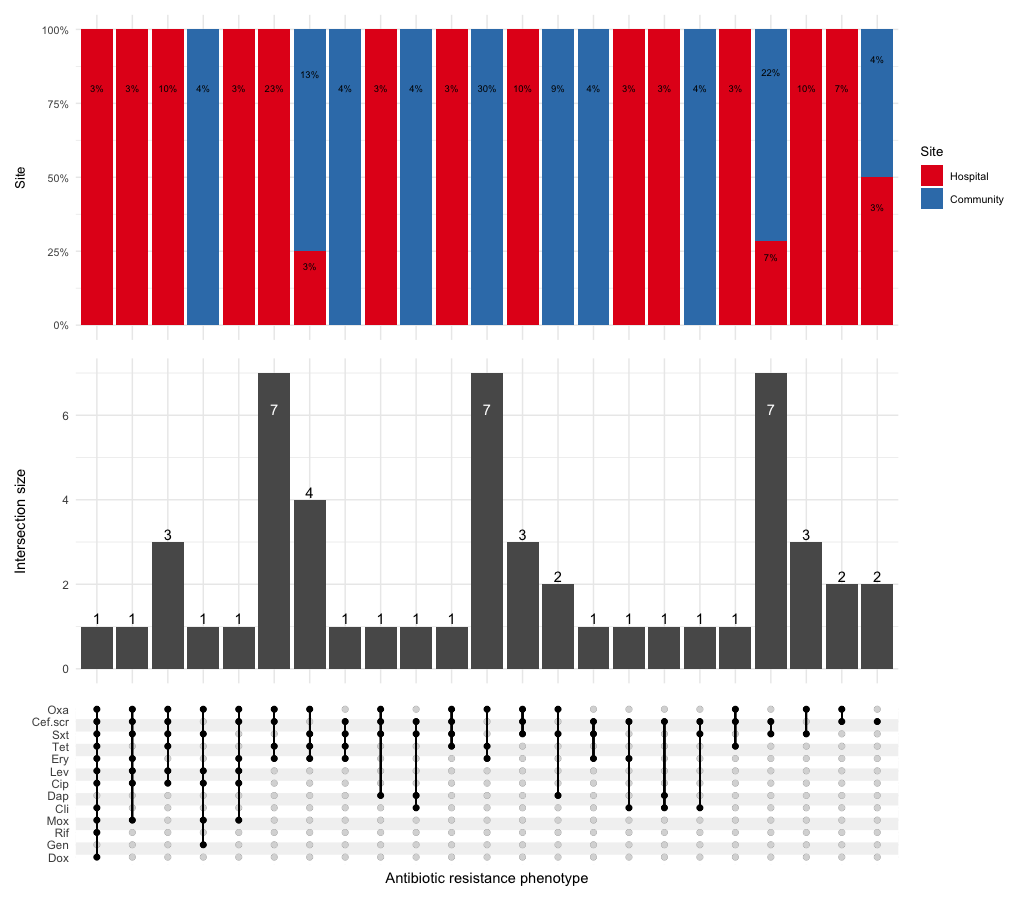 |
| --- |

**Supplementary Fig. S5**. **Distribution of MRSA antibiotic resistance phenotypes**. Lower panel shows MDR intersections for all isolates (n = 53) with one or more antibiotics. The bars show the number of isolates with the depicted MDRO phenotype (intersection). Top figure shows the percent distribution of MRSA antibiotic resistance phenotypes for isolates from hospitals (n = 30) and communities (n = 23). Antibiotics: oxacillin (Oxa), cefoxitin screen (Cef.scr), sulfamethoxazole+trimethorim (Sxt), tetracycline (Tet), erythromycin (Ery), levofloxacin (Lev), ciprofloxacin (Cip), daptomycin (Dap), clindamycin (Cli), moxifloxacin (Mox), rifampicin (Rif), gentamicin (Gen), doxycycline (Dox).
